# HIV-1 Protease and Reverse Transcriptase Control the Architecture of Their Nucleocapsid Partner

**DOI:** 10.1371/journal.pone.0105187

**Published:** 2014-08-05

**Authors:** The PLOS ONE Staff

Animation S1 and Animation S2 in the Supporting Information section do not open correctly. The authors have provided accessible versions below.

## Supporting Information

Animation S1 An animated model of HIV-1 reverse transcription. In order to better understand the overall process of HIV reverse transcription, an animated model is available here for downloading. Animation S1 is a MOV file for viewing with Apple Quicktime and Animation S2 is a SWF file for viewing with the Flash plugin of Microsoft Internet Explorer or Mozilla Firefox web browsers. Note that with the MOV file (S2) the space bar is available to pause and to start the animation. This animation is derived from the classical model of reverse transcription [A. Telesnitsky and S. P. Goff, Reverse transcriptase and the generation of retroviral DNA, in Retrovirus, J. M. Coffin, S. H. Hughes and H. E. Varmus Eds, (1997) CSHL Press]. For clarity, this animation represents an idealized process with a figure-of-eight-shaped nucleic-acid template. Attention has been focused on the following points: the three primers (tRNALys3, 3′ppt and cppt) are oversized for clarity; the two RNA ends (RU5 and U3R) are in close proximity throughout reverse transcription, in order to show a continuous process at the time of the strand transfers; the RT-associated RNAseH is shown with a high activity for digestion of the RNA following minus strand DNA elongation. The two polypurine tracts are presented as the two exclusive primers for plus strand DNA initiation, whereas the remaining RNA fragments are proposed to be displaced by the two translocating RT molecules during minus strand DNA elongation. The central DNA flap is viewed as a fluctuating structure, the function of which still needs to be elucidated. The presentation of the timing for plus strand DNA termination in combination with the central flap synthesis and LTR duplication seems the most representative to the authors. This model does not exclude alternative pathways, especially considering the dimeric nature of the initial RNA template. (0.13 MB MOV)
(MOV)

Animation S2 See Legend of Animation S1. (0.13 MB SWF)
(SWF)
